# New Mydriasis-Free Electroretinogram Recorded with Skin Electrodes in Healthy Subjects

**DOI:** 10.1155/2017/8539747

**Published:** 2017-06-20

**Authors:** Ken Asakawa, Kana Amino, Machiho Iwase, Yuki Kusayanagi, Akiho Nakamura, Rio Suzuki, Takashi Yuuki, Hitoshi Ishikawa

**Affiliations:** Department of Orthoptics and Visual Science, School of Allied Health Sciences, Kitasato University, Kanagawa, Japan

## Abstract

**Purpose:**

To evaluate the reproducibility and consistency of the new mydriasis-free electroretinogram (ERG) with a skin electrode (RETeval) device, to determine the normative values of parameters, and to clarify the usefulness of pupil records to colored-light stimulus.

**Methods:**

A total of 100 eyes of 50 healthy subjects (mean age, 21.4 years) were enrolled. The diagnostic parameters obtained by the RETeval device were examined under the following conditions. The reproducibility was determined with the coefficient of variation (CV). The consistency was examined by intraclass correlation coefficients (ICCs). The mean value and the normal range were analyzed with a 95% confidence interval as the normative values of parameters. The correlation of parameters to pupil records (area ratio, constriction ratio) and flicker ERG was also examined in the diabetic retinopathy assessment protocol.

**Results:**

From the CV for each of the two measurements, the amplitude has a low reproducibility compared with the implicit time. Generally good consistency was obtained with both ERG parameters (ICCs = 0.48–0.92). Moderate correlations were found for the white-, red-, and blue-light stimulus in the area ratio and the constriction ratio, respectively (*r* = 0.44–0.62; *P* = 0.010–<0.0001). No correlation was observed between pupil and flicker parameters (*r* = 0.06–0.34; *P* = 0.646–0.051).

**Conclusions:**

The RETeval device was suggested as a possible screening device to detect the visual afferent diseases by evaluating in combination with the ERG recording and the colored-light pupil response.

## 1. Introduction

The electroretinogram (ERG) has been commonly used to distinguish various retinal diseases and evaluate their stages in clinical practice [1]. However, it requires the technical proficiency of a trained examiner. The RETeval device (LKC Technologies Inc., Gaithersburg, USA), which has recently become commercially available, employs a protocol capable of recording 5 types of signals (cone, flicker, rod, maximal, and oscillatory potentials) without dilating eye drops [2]. This device is easy to use (width 7 cm × depth 10 cm × height 23 cm; weight 232 g). It is especially effective with its characteristics that employ skin electrodes, which pose no risk of corneal damage or infection [2–7]. The RETeval device has the additional merits that it is capable of simultaneous recording of flicker-responses and pupil area ratio, namely, pupil light responses, by using the diabetic retinopathy (DR) assessment.

On the other hand, the determination of the normative values of healthy subjects is of utmost importance because there are no normative values of ERG parameters such as implicit time (msec) and amplitude (*μ*V). Moreover, the significance of the pupil parameters has not yet been clarified.

The purposes of the present study were as follows: first, to evaluate the reproducibility of the records with an examiner and the consistency of the records between examiners regarding parameters obtained by the RETeval device; second, to determine the normative values; and, third, to clarify the usefulness of pupil records.

## 2. Materials and Methods

### 2.1. Subjects

We examined 100 eyes of 50 healthy subjects (12 males and 38 females), ranging in age from 20 to 24 years old (21.4 ± 0.9, mean ± SD). The inclusion criteria required a visual acuity of 20/20, degree of myopia ≤−10.00 D, and a pupil size of at least 4.0 mm without dilation to allow good-quality waves to be obtained. Written informed consent was obtained from all the subjects after explanation of the purpose and procedure of the study. This study was approved by the Ethics Committee of Kitasato University, School of Health Allied Sciences (2016-G023B).

### 2.2. ERG and Pupil Recordings

ERG and pupil recordings were performed during the period from 10 AM to 2 PM, when the condition of the pupil is most stable, from the right to the left eye in a sitting posture. The skin electrode was placed on the orbital rim 2 mm from the margin of the lower eyelid, and the eyes were examined by directing the subject's gaze at the red fixation spot in the center of the eyecup, with first the left and then the right eye covered with the subject's hand.

After 20 minutes of light adaptation, the cone-system response was evaluated by the records of response in the order of cone-response to flicker-response. By using the protocol of the DR assessment [4], the flicker-response (luminance of 16 and 32 Td-s) and pupil response (luminance of 4 and 32 Td-s) to 5-second stimulus with white, red (621 nm), and blue light (470 nm) were simultaneously recorded. Then, after 20 minutes of dark adaptation, the rod-system response was evaluated by the records of responses from rod (scotopic b-wave)-response to maximal-response to oscillatory potentials (sum).

The retinal illuminance energy of cone-response, flicker-response, and maximal-response was 85 Td-s, and the only rod-response was 0.24 Td-s. The background illuminance under the light adaptation was 848 Td-s. The stimulus frequency was 0.5 Hz for 9 times for the rod-response, 2 Hz for 30 times for the cone-response, 28.3 Hz for 141–424 times for the flicker-response, and 0.1 Hz for 5 times for the maximal-response. The oscillatory potential waveform is obtained by applying 85–190 Hz bandpass filter (to the maximal-response). Then, up to 5 cursors are automatically placed on the oscillatory potential peaks and troughs and are indicated on the report as black dots on the waveform by device software. Implicit times (time to peak) and amplitudes (peak to following trough) are reported for each individual cursor. The sums of implicit times and amplitudes for all cursors are also reported.

The signal processing for the flicker tests has previously been described in detail [2]. Contrastingly, that for the nonflicker tests uses the followings steps. A zero-phase 0.3 Hz high-pass filter reduces electrode drift and offset while preserving waveform timing. Measurements from multiple flashes are combined to improve the signal-to-noise ratio. The resulting waveform is then processed using wavelet-based denoising, where wavelets are attenuated based on the signal-to-noise power between the poststimulus (signal) and prestimulus (noise) portions of the waveform [8].

These red and blue-light sources used for pupil response measurements, which are feasible for colored-light stimulus under a custom protocol for research purposes, were manufactured by LKC Technologies Inc. and ordered from the MAYO Corporation (Aichi, Japan).

### 2.3. Analysis

Statistical analysis was performed using commercially available statistical software (SPSS, version 20.0; IBM Corporation, Armonk, NY).

#### 2.3.1. Intraexaminer Reproducibility

Reproducibility of parameters obtained by the measurement of the right eyes (50 eyes) of all the subjects twice at 1-minute intervals by an examiner (examiner A) was evaluated by the coefficient of variation (CV), calculated by dividing the value of the standard deviation by the mean value.

#### 2.3.2. Interexaminer Consistency

Regarding the parameters obtained by the measurement of the left eyes (50 eyes) of all the subjects twice at 1-minute intervals, their consistency between 2 examiners (examiners B and C) was evaluated by intraclass correlation coefficients (ICCs). Consistency was expressed as “almost perfect” when ICC was ≥0.81, as “substantial” when it was 0.80–0.61, and as “moderate” when it was 0.60–0.41.

#### 2.3.3. Normative Values of Parameters

The normative values of ERG parameters were calculated from the mean of two consecutive measurements obtained by the same examiner (examiner A). Then, the mean value and the normal range were analyzed with the upper and lower confidence limits with a 95% confidence interval (CI).

#### 2.3.4. Pupil Response and Flicker-Response to Colored-Light Stimulus

DR assessment to colored-light stimulus was evaluated from the measurement of the right and left eyes of the subjects once at 1-minute intervals by an examiner (white-light stimulus: examiner D; red-light stimulus: examiner E; blue-light stimulus: examiner F), respectively.

Pupil response to white-, red-, and blue-light stimulus for 5 seconds each was automatically analyzed by the area ratio (difference of pupil area under luminance of 4 and 32 Td-s) and constriction ratio (under luminance of 32 Td-s) (%) that were manually calculated by the equation: (initial pupil size before light stimulus − minimal pupil size during light stimulus)/(initial pupil size before light stimulus) × 100

Pearson product-moment correlation coefficient (*r*) was used to evaluate correlation between the area ratio and the constriction ratio. The correlation of the implicit times and the amplitudes with the flicker-response and the area ratio and the constriction ratio, which are pupil parameters, were also determined. Correlation of ≥0.60 was interpreted to indicate strong correlation, and the correlation coefficients between 0.35 and 0.59 were interpreted as indicating moderate correlation. A *P* value of <0.05 was considered statistically significant.

## 3. Results

### 3.1. Intraexaminer Reproducibility of ERG Parameters

Table 1 summarizes the CVs for the ERG that were performed in 50 eyes (right eye). For the implicit time, the CVs for two repeated values determined by the same examiner were 9.5% and 9.7% for the a-wave of the cone-response, 3.8% and 4.0% for the b-wave of the cone-response, 2.6% and 2.5% for the flicker-response, 14.6% and 12.8% for the rod-response, 7.5% and 7.1% for the a-wave of the maximal-response, 11.8% and 9.3% for the b-wave of the maximal-response, and 8.5% and 3.3% for the oscillatory potentials. For the amplitude, the CVs were 37.0% and 34.9% for the a-wave of the cone-response, 33.3% and 30.2% for the b-wave of the cone-response, 30.1% and 30.3% for the flicker-response, 31.9% and 29.8% for the rod-response, 34.0% and 33.9% for the a-wave of the maximal-response, 30.9% and 32.3% for the b-wave of the maximal-response, and 40.8% and 32.2% for the oscillatory potentials. The reproducibility of the amplitude was considerably lower than the implicit time.

### 3.2. Interexaminer Consistency of ERG Parameters

A summary of the interexaminer consistency for the two examiners that performed the ERG in 50 eyes (left eye) is shown in Table 2. For the implicit time, the ICCs were 0.48 for the a-wave of the cone-response, 0.76 for the b-wave of the cone-response, 0.91 for the flicker-response, 0.77 for the rod-response, 0.51 for the a-wave of the maximal-response, 0.63 for the b-wave of the maximal-response, and 0.85 for the oscillatory potentials. For the amplitude, the ICCs were 0.71 for the a-wave of the cone-response, 0.87 for the b-wave of the cone-response, 0.92 for the flicker-response, 0.83 for the rod-response, 0.72 for the a-wave of the maximal-response, 0.92 for the b-wave of the maximal-response, and 0.87 for the oscillatory potentials. High or moderate degrees of consistency were obtained for all parameters.

### 3.3. Normative Values of ERG Parameters

**Figure 1:**
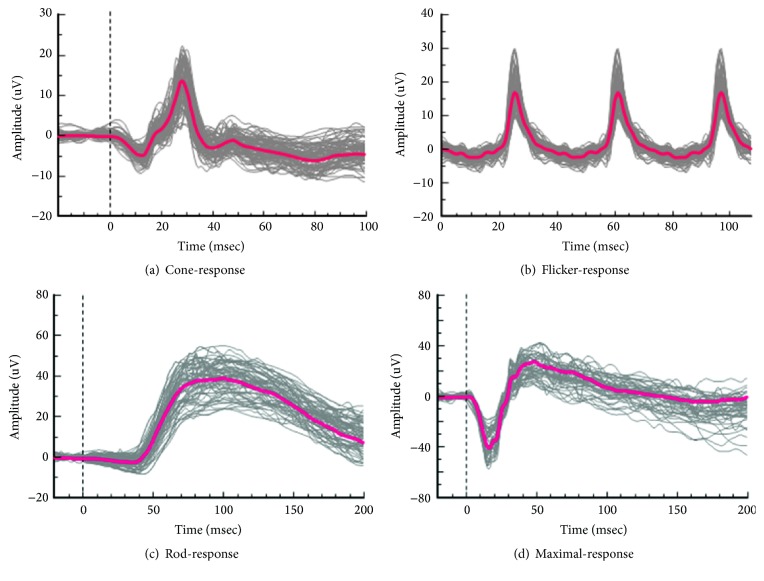
All waveforms of the 50 eyes (red line shows mean waveform).

Figure 1 shows all the waveforms that were used to evaluate the normative values of the 50 eyes (right eye).  Table 3 shows the normative values with the normal range as determined by the 95% CI of the parameters. For the implicit time (msec), the mean values were 12.1 (12.0–12.2 of normal range) for the a-wave of the cone-response, 28.2 (28.1–28.3) for the b-wave of the cone-response, 24.6 (24.6–24.7) for the flicker-response, 96.5 (95.4–97.7) for the rod-response, 14.8 (14.7–14.9) for the a-wave of the maximal-response, 46.7 (46.3–47.2) for the b-wave of the maximal-response, and 152.5 (151.8–153.2) for the oscillatory potentials. For the amplitude (*μ*V), the mean values were 5.8 (5.6–6.0) for the a-wave of the cone-response, 21.1 (20.4–21.7) for the b-wave of the cone-response, 21.6 (21.0–22.2) for the flicker-response, 44.4 (43.2–45.6) for the rod-response, 40.5 (39.3–41.8) for the a-wave of the maximal-response, 68.7 (66.7–70.6) for the b-wave of the maximal-response, and 49.0 (47.3–50.6) for the oscillatory potentials.

### 3.4. Pupil Response and Flicker-Response to Colored-Light Stimulus

A summary of the mean values and the 95% CI of the pupil parameters are shown in Table 4. The mean area ratios for the white-light stimulus, red-light stimulus, and blue-light stimulus were 2.4 (2.2–2.6), 2.3 (2.1–2.5), and 1.9 (1.8–2.1) for the right eye and 2.2 (2.1–2.3), 2.1 (1.9–2.2), and 1.8 (1.7–1.9) for the left eye, respectively. The mean constriction ratios were 37.4 (35.9–38.8), 34.3 (32.3–36.3), and 39.2 (37.9–40.6) for the right eye and 38.8 (37.6–40.0), 36.1 (34.4–37.7), and 38.6 (37.2–40.0) for the left eye. The mean DR scales were 21.2 (20.7–21.8) for the white-light stimulus, 23.0 (22.3–23.7) for the red-light stimulus, and 21.9 (21.2–22.6) for the blue-light stimulus, respectively.

**Figure 2:**
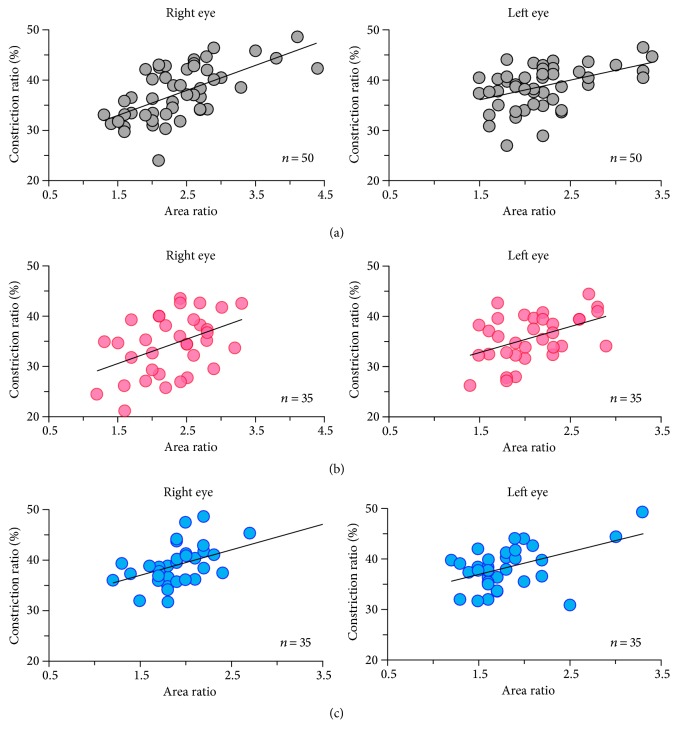
*Correlation between the area ratio and the constriction ratio*. Moderate correlations are found for the white-light stimulus (right eye *r* = 0.62, *P* < 0.0001; left eye *r* = 0.46, *P* = 0.002), the red-light stimulus (right eye *r* = 0.45, *P* = 0.008; left eye *r* = 0.44, *P* = 0.010), and the blue-light stimulus (right eye *r* = 0.53, *P* = 0.001; left eye *r* = 0.48, *P* = 0.003).

For the pupil parameters of the area ratio and the constriction ratio, moderate correlations were found for the white-light stimulus (right eye *r* = 0.62, *P* < 0.0001; left eye *r* = 0.46, *P* = 0.002), the red-light stimulus (right eye *r* = 0.45, *P* = 0.008; left eye *r* = 0.44, *P* = 0.010), and the blue-light stimulus (right eye *r* = 0.53, *P* = 0.001; left eye *r* = 0.48, *P* = 0.003) (Figure 2). For DR assessment of the flicker-response, the mean implicit times are shown in Table 5, and the mean amplitudes are shown in Table 6. No correlation was observed between pupil and flicker parameters (*r* = 0.06–0.34; *P* = 0.646–0.051).

## 4. Discussion

RETeval parameters had generally good interexaminer consistency. However, the amplitude showed a generally lower intraexaminer reproducibility compared to the implicit time. The possible hypotheses include the pupil size recovery and adaptation of photoreceptor cells after a flash stimulus [9]. There should be an interval of at least a few minutes between stimulus flashes. Kato et al. [2] reported that a factor affecting the amplitude and implicit time in the flicker-response was pupil size, whereas Miura et al. [5] mentioned it was the presence of Grade 2 or higher cataracts in the Emery-Little classification. One of the aims of the present study was to investigate and compare the normative values of young subjects in their twenties by taking advantage of the RETeval device with which mydriasis-free evaluation is possible. Therefore, the normative values of people older than thirty years remain unknown. In the future, these evaluations should be included to address this limitation. While the factors of aging and gender should be taken into consideration, the normative values as “reference levels” may be useful for distinguishing various retinal diseases and evaluating the stages of those diseases in clinical practice.

For the DR assessment protocol, although it is well known that DR scales are associated with the severity of diabetic retinopathy [7], the usefulness of the area ratio has not yet been clarified. In the present study, for the pupil parameters of the area ratio and the constriction ratio, moderate correlations were found; however, no correlation was observed between the pupil parameters and the amplitude and implicit time in flicker-response. It is known that pupil response to light stimulus is impaired in optic nerve diseases. Accordingly, a differential diagnosis between diseases of the optic nerve and those of the retinal photoreceptor cells might be possible by the impairment of the pupil constriction and by the aberrant values of amplitude and implicit time in the ERG response. Therefore, ERG and pupil parameters with RETeval are good markers to judge whether visual dysfunction is ascribable to the lesion in the retina itself or that in the afferent pathway such as the optic nerve.

Retinal photoreceptor cells were regarded as rods and cones for many years; however, Provencio et al. [10] isolated melanopsin, a novel visual pigment, and reported that melanopsin was identified in the retinal ganglion cells of the inner retina. The melanopsin-containing retinal ganglion cells (mRGCs) respond to depolarization regardless of visual input from conventional photoreceptors of the outer retina, and they have projections to the olivary pretectal nucleus and Edinger-Westphal nucleus in the midbrain and play a role in the pupil light response [11, 12]. For these photoreceptions by mRGCs based on wavelength characteristics that lead to pupil constriction in monkeys [13], humans [14], and rabbits [15] with complete retinal photoreceptor loss or dysfunction, sensitivity is highest for 460–480 nm blue-light stimulus. The colored-light pupil response may be used to predict the retinal state in patients with the outer or inner retina diseases.

One difficulty encountered during the study was the lack of availability of a custom protocol, which resulted in the curtailment of the acquisition of a lot of pupil data. We could not evaluate the intraexaminer reproducibility and interexaminer consistency for pupil parameters. However, the device is capable of simultaneous recording of flicker-responses and pupil responses using the protocol of the DR assessment. Therefore, we considered that the reproducibility and consistency for pupil parameters are consistent with the flicker-response parameters.

If the diseases of the optic nerve or retina are discriminatively detected by ERG recording with skin electrodes in combination with the records of pupil response to colored-light stimulus, not only would the burden of unnecessary ophthalmologic tests for patients be reduced but also this most noninvasive simple estimation method would contribute to the early detection of eye diseases.

Although the present study needs a larger range of subjects of different ages, the normative data could be evaluated in combination with those in the pupil response to colored-light stimulus. Consequently, the RETeval device was suggested as a possible screening device to detect signs and symptoms in visual afferent systems.

## Figures and Tables

**Table 1:** Reproducibility of ERG parameters.

ERG parameters		Implicit time (ms)		Amplitude (*μ*V)	
		CV		CV	
		Test	Retest	Test	Retest
Cone-response	a-wave	9.5	9.7	37.0	34.9
	b-wave	3.8	4.0	33.3	30.2
Flicker-response 28.3 Hz		2.6	2.5	30.1	30.3
Rod-response	Scotopic b-wave	14.6	12.8	31.9	29.8
Maximal-response	a-wave	7.5	7.1	34.0	33.9
	b-wave	11.8	9.3	30.9	32.3
Oscillatory potentials		8.5	3.3	40.8	32.2

CV: coefficient of variation.

**Table 2:** Consistency of ERG parameters.

ERG parameters		Implicit time (ms)	Amplitude (*μ*V)
		ICC	ICC
Cone-response	a-wave	0.48	0.71
	b-wave	0.76	0.87
Flicker-response 28.3 Hz		0.91	0.92
Rod-response	Scotopic b-wave	0.77	0.83
Maximal-response	a-wave	0.51	0.72
	b-wave	0.63	0.92
Oscillatory potentials		0.85	0.87

ICC: intraclass correlation coefficients.

**Table 3:** Normative values of ERG parameters.

ERG parameters		Implicit time (ms)		Amplitude (*μ*V)	
		Mean	Range (95% CI)	Mean	Range (95% CI)
Cone-response	a-wave	12.1	12.0–12.2	5.8	5.6–6.0
	b-wave	28.2	28.1–28.3	21.1	20.4–21.7
Flicker-response 28.3 Hz		24.6	24.6–24.7	21.6	21.0–22.2
Rod-response	Scotopic b-wave	96.5	95.4–97.7	44.4	43.2–45.6
Maximal-response	a-wave	14.8	14.7–14.9	40.5	39.3–41.8
	b-wave	46.7	46.3–47.2	68.7	66.7–70.6
Oscillatory potentials		152.5	151.8–153.2	49.0	47.3–50.6

CI: confidence interval.

**Table 4:** Pupil response of DR assessment to colored-light stimulus.

DR assessment		Pupil response					
		White		Red		Blue	
		Mean	Range (95% CI)	Mean	Range (95% CI)	Mean	Range (95% CI)
AR	RE	2.4	2.2–2.6	2.3	2.1–2.5	1.9	1.8–2.1
	LE	2.2	2.1–2.3	2.1	1.9–2.2	1.8	1.7–1.9
CR	RE	37.4	35.9–38.8	34.3	32.3–36.3	39.2	37.9–40.6
	LE	38.8	37.6–40.0	36.1	34.4–37.7	38.6	37.2–40.0
DR		21.2	20.7– 21.8	23.0	22.3–23.7	21.9	21.2–22.6

CI: confidence interval, AR: area ratio, CR: constriction ratio, and DR: diabetic retinopathy scale.

**Table 5:** Implicit time of DR assessment to colored-light stimulus.

DR assessment		Implicit time (ms)					
		White		Red		Blue	
		Mean	Range (95% CI)	Mean	Range (95% CI)	Mean	Range (95% CI)
16 Td-s	RE	26.8	26.5–27.2	29.1	28.7–29.6	26.1	25.7–26.5
	LE	26.7	26.4–27.1	28.5	28.1–29.0	26.2	25.8–26.6
32 Td-s	RE	26.1	25.8–26.3	27.5	27.1–27.9	25.7	25.3–26.1
	LE	26.0	25.8–26.2	27.1	26.8–27.4	26.1	25.8–26.4

CI: confidence interval.

**Table 6:** Amplitude of DR assessment to colored-light stimulus.

DR assessment		Amplitude (*μ*V)					
		White		Red		Blue	
		Mean	Range (95% CI)	Mean	Range (95% CI)	Mean	Range (95% CI)
16 Td-s	RE	16.8	15.3–18.3	16.5	15.0–18.0	20.2	18.7–21.7
	LE	14.7	13.2–16.2	14.1	12.5–15.8	18.1	16.5–19.8
32 Td-s	RE	20.9	19.0–22.7	21.0	19.1–22.9	22.0	20.1–23.9
	LE	18.1	16.5–19.7	17.8	15.9–19.6	19.6	17.7–21.4

CI: confidence interval.
